# Efficacy of Handheld Ultrasound in Medical Education: A Comprehensive Systematic Review and Narrative Analysis

**DOI:** 10.3390/diagnostics13243665

**Published:** 2023-12-14

**Authors:** Mariam Haji-Hassan, Roxana-Denisa Capraș, Sorana D. Bolboacă

**Affiliations:** 1 Department of Medical Informatics and Biostatistics, Iuliu Hațieganu University of Medicine and Pharmacy, Louis Pasteur Str., No. 6, 400349 Cluj-Napoca, Romania; mariam.haji-hassan@umfcluj.ro (M.H.-H.); sbolboaca@umfcluj.ro (S.D.B.); 2 Department of Anatomy and Embryology, Iuliu Hațieganu University of Medicine and Pharmacy, Clinicilor Str., No. 3–5, 400006 Cluj-Napoca, Romania

**Keywords:** handheld ultrasound, medical students, ultrasound training, medical education

## Abstract

Miniaturization has made ultrasound (US) technology ultraportable and, in association with their relatively low cost, made handheld devices attractive for medical education training programs. However, performing an ultrasound requires complex skills, and it is unclear whether handheld devices are suitable for the training of novices. Our study aimed to identify to what extent handheld US devices can be employed in medical undergraduates’ and residents’ education. We selected studies that evaluate the results obtained by students and/or residents who have participated in ultrasound training programs using handheld devices. The studies were included if they reported post-test (pre-test optional) achievements or a comparison with a control group (a group of experts or novices who underwent a different intervention). Twenty-six studies were selected, and their characteristics were summarized. Handheld ultrasound devices were used in training programs to learn echocardiography, abdominal, and/or musculoskeletal ultrasound. Statistically significant progress was noted in the ability of naïve participants to capture and interpret ultrasound images, but training duration influenced the outcomes. While ultrasound training using handheld devices has proven to be feasible for various body regions and purposes (e.g., better understanding of anatomy, clinical applications, etc.), the long-term impacts of handheld education interventions must be considered in addition to the short-term results to outline guidelines for targeted educational needs.

## 1. Introduction

Reducing the size of medical equipment has become the norm, and size decreases are usually accompanied by a decrease in price [1]. Ultrasound (US) probes can be carried in a lab coat and connected to smartphones and tablets, all for a fraction of their larger counterparts’ costs [2]. Their increased accessibility and high portability make them attractive for medical education and general practice alike [3,4]. No recommended set dimensions and weights existed for ultraportable US devices until 2012, when the World Health Organization provided an expected dimension (13 × 7 × 1 cm), weight (300 g), and price range (between USD 5000 and USD 10,000) [5]. However, the prices of handheld US devices ranges from around USD 3000 (USD 2699 for one probe + USD 420/yr membership for Butterfly iQ [6]) to around USD 9300 (Kosmos Torso-One [7]). The brands available on the market exhibit specific features and are superior in terms of specific characteristics (e.g., Lumify™ has the highest image quality, and Vscan Air™ is the best in terms of ease of use) [8].

Handheld ultrasound devices can be vital in developing countries and other resource-limited settings, often being the only available imaging devices. Ultraportable devices have already been used in emergency medicine [9,10], remote areas [11], disaster responses (e.g., the New Mexico Disaster Medical Assistance Team used handheld ultrasound machines to aid patient care, and this proved effective in making management decisions [12]), and military situations (wherein they are easy to use, valuable in discerning a diagnosis [13]), or they can act as an auxiliary to clinical examination at point-of-care examination [14,15,16,17].

Ultrasound is a complex imaging modality, and image capturing and image interpretation require training [18,19], but there are still no standardized approaches regarding the content of training programs, their duration, or required trainer qualifications [20]. Considering that ultrasound examinations are user-dependent, the results reported in the scientific literature support the accuracy in measurements and lower image quality when compared to conventional US cardiac examinations [21,22,23]. 

Handheld ultrasound devices are finding a place in medical students’ pockets. Irvine Medical School (University of California) offered all first-year students a Butterfly iQ ultrasound probe [24]. Handheld ultrasound technology has proven to be a valuable adjunct to physical examinations in experienced hands, despite the lower resolution of the images involved [25]. For an inexperienced student or resident, lower image quality is more likely to impact their ability to make a correct diagnosis. On the other hand, the increased accessibility of handheld devices could make up for their downsides. In 2017, Galusko et al. [26] reported on the use of handheld ultrasound devices in medical education and concluded that data are scarce and incomplete. Considering technological developments, in this study, we aim to provide an update on the state of the art regarding the use of handheld US training in medical education, both for undergraduate medical students and residents. 

## 2. Materials and Methods

Our systematic review was performed following the PRISMA 2020 (Preferred Reporting Items for Systematic Reviews and Meta-Analyses) guidelines [27]. The protocol of the review was registered on PROSPERO (CRD42021249070, first search). 

### 2.1. Search Strategy

The search strategy was constructed to address the following:

- Problem: medical students and residents lack of competence in performing ultrasounds.
- Intervention: ultraportable (handheld) ultrasound-based training program.
- Comparisons: pre- and post-tests given to study subjects; comparison between two groups (experimental group: handheld training vs. a group of experts or a group of novices who underwent a different intervention or no intervention).
- Outcome: skills in acquiring and interpreting ultrasound images.
- Study design: any exceptional case reports.

Our initial search of the literature was carried out on 15 May 2021. We performed a literature search on PubMed, ScienceDirect, and PMC using the following search string: (portable OR handheld OR pocket-size) AND ultrasound AND (education OR training OR students OR residents).

A second search was conducted with the same search string on 23 November 2023 to cover the latest studies reported in the scientific literature.

We selected studies that used a truly handheld ultrasound device in an ultrasound training program aimed at medical students and/or residents. We excluded studies that used any other portable ultrasound devices (e.g., laptop-size devices); studies involving training medical specialists instead of students and residents; secondary research papers (e.g., reviews, systematic reviews, meta-analyses); letters to editors; and case reports.

We collected information regarding the aim of the study, the type of ultraportable device that was used, the anatomical structures or pathologies that were assessed, the number of students or residents involved, the type of training program and its duration, the number of patients that were scanned, and data reflecting the performance of the educational intervention. 

### 2.2. Article Selection

The selection of the articles involved a two-step screening process. The screening process was carried out after the elimination of duplicates using the *Duplicate values …* feature in Conditional Formatting (Excel, Microsoft Office 365), applied to title and/or identification numbers (DOI, or PMID, or PMCID). Two independent researchers screened all articles’ titles and abstracts for relevancy to handheld devices (first step). The full texts were retrieved in the second step, and two independent researchers assessed the studies for inclusion. Consensus was mediated by a senior researcher to settle any disagreements.

### 2.3. Data Extraction

A self-developed extraction form was used for data extraction. The following information was collected independently by two researchers: (1) study settings (e.g., when?, where?, duration?, etc.); (2) study design (e.g., type, target condition, study aim, portable device used, participants); (3) study results (e.g., evaluated subjects, protocol and period of training, parameters used for the evaluation of trainees).

### 2.4. Quality of Reporting Assessment

The MERSQI tool [28], developed to appraise methodological quality in medical education research, was used to evaluate the quality of the studies.

### 2.5. Presentation of the Findings

A narrative method was used to synthesize the results, including a description of the study selection process, a summary of the characteristics of the included studies, an overview of the various study strategies employed, and conclusions.

## 3. Results

**Figure 1:**
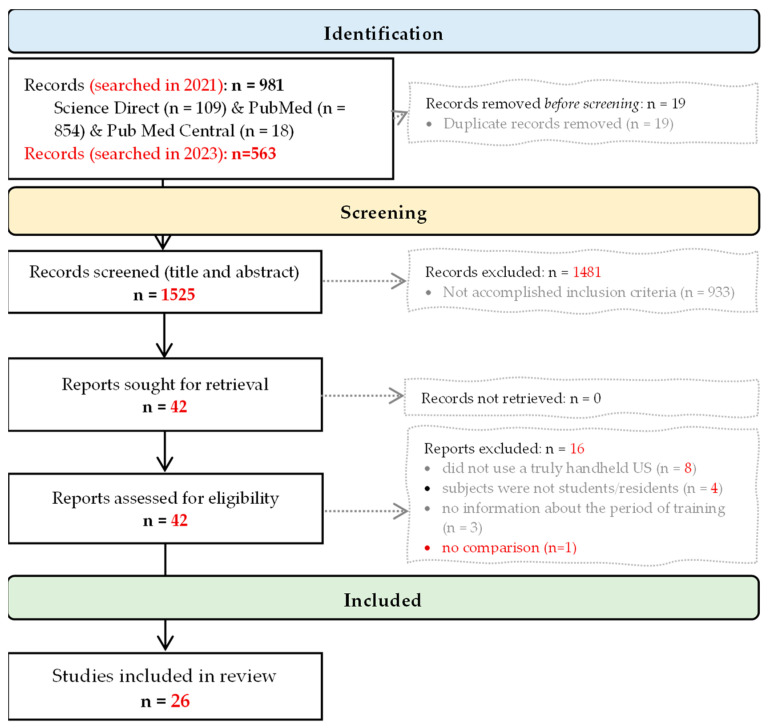
PRISMA flow diagram (from records identification to inclusion).

The search yielded 1525 articles after the removal of duplicates. A total of 42 full-text studies were assessed for eligibility, and 26 were selected and summarized (Figure 1).

### 3.1. The Trainees

Out of 26 articles, the majority provided educational interventions to medical students (Gogalniceanu et al., 2010 [29]; Swamy et al., 2012 [30]; Bonnafy et al., 2013 [31]; Cawthorn et al., 2014 [32]; Andersen et al., 2014 [33], Stokke et al., 2014 [34], Yan et al., 2015 [35]; Kapur et al., 2016 [36]; Yan et al., 2018 [37]; Galusko et al., 2018 [38]; Jerg et al., 2018 [39]; Nausheen et al., 2019 [40], Kameda et al., 2020 [41], Nausheen et al., 2020 [42], Kaiser et al., 2022 [43], Jujo et al., 2022 [44], Slader et al., 2022 [45], Edwards et al., 2023 [46], Bui et al., 2023 [47]) (see Table 1, Table 2 and Table 3). Six studies involved residents (Wilkinson et al., 2014 [48], Andersen et al., 2015 [49], Maetani et al., 2018 [50], Bar et al., 2021 [51], Acheampong et al., 2022 [52]), and two studies included both students and residents (Panoulas et al., 2013 [53]; Mai et al., 2013 [54]). The year of the study was not generally reported for residents [48,49,50,51,52,53,54]. Prior to the intervention, participants had limited experience or no experience in carrying out ultrasounds (either conventional or handheld ones). 

### 3.2. The Handheld Ultrasound Devices

General Electric’s Vscan was exclusively used in 11 studies [31,32,33,34,35,36,37,38,39,41,43,49,51,53,54]. Two studies used the Acuson P10 (Siemens, [29,54]), two used the Philips Lumify [50,52], three used the Butterfly iQ [44,45,47], and one used the Ballater Medical 3-in-1 ultrasound probe (Ballater Medical, Edinburgh, UK) [46]. Four articles did not mention the brand of the portable ultrasound device used during the training process [30,40,42,48]. 

### 3.3. Training

The training programs ranged from roughly 30 min [30] to up to 8 weeks [32], and all programs offered hands-on training. The minimum amount of time dedicated to practical training per student was 10-15 min [30], while other programs offered unlimited access to simulators/handheld devices for the entire duration of the training (up to 4 weeks) [32]. Two studies provided unlimited access to learning resources, implying a partly self-directed framework [32,50]. In some studies, the training was completely self-directed [41,50].

### 3.4. Outcomes

The heterogeneity of the measured outcomes is a characteristic of the evaluated studies, but most of them targeted or included the cardiac system (Table 3). Some studies reported a significant increase in diagnostic accuracy when handheld ultrasound technology was used in concert with a physical examination [35,39,53].

Some contradictory results have been noted in the evaluated articles. For example, two articles reported adequate imaging and measurements of the abdominal aorta diameter by students in more than 92% of cases [31,33], with no learning curve noted over time [31], while only 50% of residents adequately imaged the abdominal aorta [49].

One study reported a higher rate of false positives after training, explaining that a short training duration is most probably insufficient [48]. Self-directed learning is reported to be effective in acquiring a theoretical knowledge of ultrasounds, whereas practical skills appear to be more effectively gained with the help of an expert sonographer [32].

Almost 90% of students consider handheld ultrasound devices helpful for improving clinical examination skills [39], 98% would like to see the continuation of ultrasound training programs in their subsequent university years [36], and 94.74% of residents agreed that access to a handheld ultrasound device improves the ability to perform imaging exams [50]. When given a choice, 96% of the students stated that they would prefer to use conventional ultrasound technology over handheld ultrasound technology due to the higher image quality and more intuitive user interfaces involved [29], while the medical image quality scores proved significantly lower in HHUS group [45]. The majority of participants felt comfortable learning the new technology (95.3% [46]), enjoyed learning abut anatomy (94.4%), and felt they gained a better understanding of the clinical relevance of learning about anatomy (97.2%) [46]. Students also reported that “it would be useful to have more ultrasound session” [42], and the majority believed that US training should be in the curriculum [46].

Edwards et al. [46], on their study on using handheld US technology in anatomy training, reported a thematic analysis of the three dimensions of the barriers in US training: image interpretation (lack of familiarity, small screen, hard to achieve a clear image, etc.), future enhancement (more background knowledge, longer or more sessions, larger screen, independent learning, etc.), and religious barriers (contact with the opposite sex and modesty concerns).

### 3.5. Medical Education Research Study Quality Assessment 

Most of the evaluated studies applied a pre-test and post-test study design, objectively measuring the participants’ knowledge and going beyond statistical analysis, but none described the validity of the applied evaluation instruments (Table 4). 

## 4. Discussion

Handheld ultrasound training has proven feasible for various body regions and purposes (e.g., by providing a better understanding of anatomy, increased diagnostic accuracy when used in concert with a physical examination, etc.) with or without assistance in the identification of organs. However, when available, a conventional high-end ultrasound device was preferred, while the displaying of US images on a small screen was reported as a barrier of handheld ultrasound examinations.

### 4.1. Rethinking Medical Education

The development of ultrasound technologies changes diagnoses (including ultrasound-guided punctures) and treatment in healthcare (e.g., interventional ultrasound). Ultrasound devices come in different sizes (from handheld devices to high-end devices), and prices (starting from USD 3000 in the case of handheld devices [6]) and features (e.g., image quality, ease of use, portability, costs, and probes) vary [8]. Nielson et al. [55] highlighted the need to define the “minimum requirements for equipment, education, training, and maintenance of skills” for all medical specialists and medical students. Given the limited number of high-end US devices, handheld US devices could be an alternative for entry-level medical US training. It has already been demonstrated that short-term (a few hours to a few days) handheld ultrasound training can significantly improve students’ and residents’ abilities to capture and interpret specific ultrasound images accurately [31,33,34,35,41,42,44]. Limiting the number of trainees in a group and increasing the number of procedures per trainee improve performance [38]. Short-duration courses might increase the number of false positive diagnoses, highlighting the importance of sufficient practice [48]. 

Regardless of the dimension, the US examination had some advantages compared to other imaging techniques, such as lack of radiation, low cost, reproducibility, and the possibility to examine patients at point-of-care [56]. The trainees preferred a conventional high-end ultrasound device when available [29], while generally, the measurements were concordant [23,45,57], and the difficulty of US learning and comfort levels were similar (*p* > 0.15) [45].

Training to acquire appropriate images and interpret the images is a must, but the optimal duration and frequency of continuous hands-on sessions still need to be determined. It may be unrealistic to expect long-lasting progress after a few hours of participation in training programs. The performances of the students in terms of identifying anatomical structures via ultrasound significantly drop at medium follow-up (6 months) in the absence of repetitions [44,58]. In ultrasound training, the consistency of the educational programs is essential, especially as their benefit has been shown to wane over time as ultrasound skills can diminish due to a lack of practice. For example, the expected number of (conventional) US examinations and interpretations in the case of focused cardiac ultrasound and transthoracic echocardiography needed to obtain accreditation depends on the level of certificate (e.g., having performed from 20 to 250 exams and up to 300 interpretations [59]). 

Hellmann et al. [60] evaluated the learning curve of thirty medical residents participating in an echocardiography training program and reported an overall technical proficiency skill improvement of 0.79 points (95% confidence interval [CI] 0.53–1.04, 0–3 assessment scale) per 10 scans completed. Interpretation accuracy also improves with an increase in the number of examinations (1.01 points, 95% CI 0.69–1.39 per 10 scans, considering a 0–3 accuracy index) [60]. Considering that ultrasound training programs for specialists take about eight weeks of intense training for the main systems (e.g., cardiac, vascular, abdominal, musculoskeletal), four weeks of basic ultrasound learning and four weeks of advanced ultrasound learning, with practical hands-on sessions lasting for several hours a day (i.e., a more extensive training program) would likely be more effective.

Radiologists’ views regarding ultrasound teaching might differ from the opinions of clinicians. Webb et al. [19] highlight that teaching students a few ultrasound basics would instill a false sense of confidence in their skills, considering the extensive training required to perform an ultrasound at the level of a board-certified radiologist. Webb et al. [19] also noted that no established systems ensure minimum competency or examination quality, even though students and medical administrators demand the inclusion of ultrasound training in medical school curricula [42,46]. 

Compared to physical examination alone, handheld ultrasound has been repeatedly proven to increase physicians’ ability to establish correct diagnoses [51,61,62]. A continuous approach to training should be employed to prevent the loss of knowledge and improve image acquisition and interpretation skills. At Loma Linda University, ultrasound education programs start in the first year of study and continue throughout the four years of the undergraduate medical curricula [63]. Dinh et al. [64] showed that ultrasound teaching during the first year of medical school significantly improves student skills and emphasizes the importance of early implementation during medical education when comparing the performances of US-trained students with those of untrained students.

Potential problems that can arise concerning ultrasound training programs are tutors having insufficient experience, a lack of established structure for the training programs, time-limited instructions, and an insufficient number of ultrasound devices (either handheld or conventional). The use of handheld devices for training novices (e.g., students and inexperienced physicians) can be more challenging due to the lower quality of the images involved [28,46,48]. The most important characteristics of handheld ultrasound devices are image quality, ease of use, portability, total costs, and the availability of different probes [8]. Image quality differences exist between different handheld probes and targeted examinations [8,65]. It should be noted that the differences in image resolution, detail, and quality vary according to the conventional US device used and user experience [66,67,68].

### 4.2. Handheld Ultrasound Devices in the Future

Rapid technological advancements and innovations will probably lead to an improvement in the quality of the images associated with handheld ultrasound devices, eventually causing the quality of these images to be close to that of the images obtained using high-end US devices. This expectation is supported by the appearance of ultrasound-on-a-chip- and silicone-chip-based handheld ultrasound probes [69], with an increase in imaging quality being expected roughly every two years. Augmented reality and extended reality algorithms are expected to be implemented to assist the user in patient positioning and guide the acquisition of images as support for both training and clinical examinations [69,70]. Furthermore, diagnostic algorithms, along with light and affordable handheld devices, could make ultrasound examinations available to more patients [71], while internet connection could allow physicians to work from locations far from hospital wards (i.e., working remotely) [72]. Murray et al. [73] already reported the feasibility of virtually supervising POCUS handheld ultrasound examinations carried out by 3rd-year undergraduate medical students in four domains, namely image generation, image optimization, clinical integration, and knowledge of indications, showing the effectiveness of digital-assisted evaluation.

Ultrasound training is expected to be included in the undergraduate medical curriculum. As US technology evolves, the emergence of smaller and more affordable handheld probes with the ability to store examination data in a cloud-based open-data system is expected, facilitating real-time data analytics. 

### 4.3. Limitations

Our review was limited to studies published in English. The evaluated studies only sometimes reported the methods used to decide which students and residents should be included in their study, and mainly, participant recruitment was based on voluntary participation. Voluntary participation induces a selection bias because the participants might have a particular interest in the technology under study. Consequently, the reported results do not reflect the target population represented by undergraduate students and residents and might only apply to some students and junior doctors. The evaluated studies had distinctly different training programs and evaluation methods, and this did not allow for the quantification of the optimal duration of instruction in relation to the trainees’ performances. Furthermore, the selected studies were indexed in some specific bibliographic databases from 2010 to 2023, reflecting the temporary stalling of hands-on medical education programs. The evaluated studies’ heterogeneity in terms of training duration and targeted organs did not allow us to perform a quantitative analysis, so the presented results are only descriptive and have limited applicability.

## 5. Conclusions

Scientific data regarding the use of handheld ultrasound training involving undergraduate students and residents is limited and heterogeneous in regard to curricula, duration of training, and targeted organs and pathologies (most frequently cardiac). The students in the evaluated studies proved they can accurately examine specific organs, but the long-term impacts of handheld education interventions must be considered in addition to students’ short-term performances to outline guidelines for targeted educational needs. These guidelines must be established to ensure that trainees retain proficiency skills. Furthermore, the financial burn and associated effects must also be evaluated.

## Figures and Tables

**Table 1:** Summary of studies with residents and others as the target population, as well as what was studied, which anatomical structures or pathologies were investigated, the number of participants who underwent training, and who the US investigations were carried out on.

Author/Year [ref]	What Was Studied?	Which Anatomical Structures/Pathologies?	No. of Participants (Specialty)	US Performed on …
Panoulas et al., 2013 [53]	clinical diagnosis skills and limited HHUS training	LV (systolic dysfunction, hypertrophy), dilated RV, valvular disease, pericardial effusion, Ao	5 final-year medical std and 3 junior doctors	122 patients
Mai et al., 2013 [54]	feasibility—cardiac ultrasound exams performed by novice users	LV systolic dysfunction, LA enlargement, lung comet-tail artifacts, inferior vena cava diameter, and collapse	1 (medical std), 1 (intern), 1 (pharmacy resident)	27 subjects (22 outpatients, 5 normal volunteers)
Bonnafy et al., 2013 [31]	Ao diameter measurements—short HHUS training	abdominal Ao	n/a	56 patients
Wilkinson et al., 2014 [48]	diagnostic and technical skills with HHUS	cardiac US	24 (internal medicine)	n/a
Andersen et al., 2015 [49]	goal-directed US with HHUS; half of the randomly selected participants used HHUS	cardiac ^a^, pleural, and pericardial effusion; liver and gallbladder abnormalities; urinary system	12 residents	199 patients
Maetani et al., 2018 [50]	musculoskeletal HHUS	10 wrist structures	20 (radiology)	n/a
Bar et al., 2021 [51]	pneumothorax	lung/pleura	4 (general surgery residents)	85 patients
Acheampong et al., 2022 [52]	echocardiography	LV and RV structure and function, presence of effusion	2 (internal medicine residents) *	100 patients

HHUS = handheld ultrasound; US = ultrasound; LV = left ventricle; RV = right ventricle; LA = left atrium; Ao = aorta; std = students. ^a^ RV and LV sizes, global systolic function, valve disease, abdominal Ao diameter, inferior vena cava. * each trainee performed 50 echocardiograms and interpreted 20 echocardiograms.

**Table 2:** Summary of studies with students as the target population, as well as what was studied, which anatomical structures or pathologies were investigated, the number of participants who underwent training, and who the US investigations were carried out on.

Author, Year [ref]	What Was Studied?	Which Anatomical Structures/Pathologies?	No. of Participants and Their Year of Study	US Performed on …
Gogalniceanu et al., 2010 [29]	feasibility—basic US skills	FAST US protocol ^a^	26 (3rd and 5th)	n/a
Swamy et al., 2012 [30]	cadaveric anatomy teaching	upper and lower limbs ^b^	215 (2nd)	on each other
Cawthorn et al., 2014 [32]	focus cardiac examination	focused cardiac US	12 (1st) and 45 (3rd)	n/a
Andersen et al., 2014 [33]	effectiveness of HHUS training	LV function, pericardial effusion, lung comets, inferior vena cava diameter, hydronephrosis, gallstones, chelocystitis, bladder distension, abdominal Ao diameter, abdominal free fluid	30 (5th)	211 patients
Stokke et al., 2014 [34]	the effectiveness of HHUS to detect clinically relevant cardiac lesions	valvular disease, LV and RV dysfunction, atrial diameters, pericardial effusion, Ao diameter	21 (n/a)	72 patients
Yan et al., 2015 [35]	feasibility (4 weeks) HHUS training	valvular heart disease	4 (3rd)	49 patients
Kapur et al., 2016 [36]	HHUS in teaching clinical anatomy	liver, spleen, kidneys, gallbladder, pancreas, urinary bladder, aorta	100 (1st)	mannequins
Yan et al., 2018 [37]	focused cardiac US	valvular heart disease ^c^	10 (3rd)	107 patients
Galusko et al., 2018 [38]	cardiac HHUS training	heart anatomy and pathology	40 (n/a)	n/a
Jerg et al., 2018 [39]	HHUS as an adjuvant to physical examinations	heart and vessels, lungs and thorax, and abdomen in emergency examination	10 (n/a)	n/a
Nausheen et al., 2019 [40]	teaching and learning basic sciences and clinical skills with HHUS	localization of liver, kidneys, Morison’s pouch, gall bladder, spleen, pancreas, aorta, and IVC, Doppler scan and the FAST exam	60 (1st)	simulated patients
Nausheen et al., 2020 [42]	abdominal anatomy structures	liver, kidney, urinary bladder, inferior vena cava and aorta, spleen, gallbladder	25 (1st)	standardized patients
Kameda et al., 2022 [41]	feasibility and efficacy of self-learning and telepresence instructions	focused cardiac ultrasound	8 (3rd) +16 (4th)	on themselves
Kaiser et al., 2022 [43]	ultrasound image quality	portal vein, liver spleen, kidneys, aorta, Douglas/rectovesical space, portal vein, pancreas, FAS Trauma, hepatic artery and hepatic veins	100 (n/a)	each other
Edwards et al., 2022 [46]	teaching sessions for anatomy education	upper and lower limb musculoskeletal structures, vessels and nerves, heart, liver, gallbladder	107 (*)	each other
Jujo et al., 2022 [44]	to independently obtain basic cardiac POCUS views and to identify normal anatomic structures	heart ultrasound—cardiac POCUS	54 (1st and 2nd)	healthy volunteer
Slader et al., 2022 [45]	US fundamentals	pulmonary US, abdominal US, cardiac US, and the Focused Assessment with Sonography for Trauma (FAST)	119 (n/a)	n/a
Bui et al., 2023 [47]	urologic US	differentiating urine from peritoneal fluid, knowing the criteria for hydronephrosis, and deciding when to use catheterization over bladder POCUS volume measurement	14 (n/a)	patients

HHUS = handheld ultrasound; US = ultrasound; std = students; LV = left ventricle; RV = right ventricle; LA = left atrium; Ao = aorta; n/a = not available. * non-medical students completing a course with a focus on anatomy and physiology. ^a^ FAST (Focused Assessment with Sonography in Trauma): Morrison’s pouch, splenorenal recess, four-chamber view of the heart, bladder and pelvis; ^b^ muscles, blood vessels, tendon, nerves, bones, menisci; ^c^ Ao stenosis/regurgitation, mitral stenosis/regurgitation, tricuspid regurgitation.

**Table 3:** Description of training and presentation of key findings.

**Author, Year**	**Main Features of Training**	**Key Findings**
Wilkinson et al., 2014 [48]	Two strategies: (a) a conventional (C) ward-based (4 one-hour sessions) strategy and (b) a technology (T)-driven (simulation-based) online module-based education and virtual trainer (CAE Healthcare).	↑ correct diagnosis: C group—156% (*p* < 0.001); T group—169% (*p* = 0.001). Quality US images: C group (53.8%) vs. T group (13.6%).
Andersen et al., 2015 [49]	Four hours formal didactic lectures (anatomy, abnormalities, image acquisition). Examinations: 95 abdominal and cardiac.	A total of 83% for the acquisition and interpretation of cardiac and abdominal structures, but only 50% for the abdominal aorta. HHUS changed, verified, or added an important diagnosis in 35% of cases.
Maetani et al., 2018 [50]	Three days of independent scanning practice. Musculoskeletal US.	Correct answers: 2.5 ± 2.16 (pre-test) vs. 9.85 ± 0.37 (post-; *p* < 0.001). A total of 94.74% of residents strongly acknowledge the benefits of HHUS.
Panoulas et al., 2013 [53]	Standardized 2-h HHUS echocardiography with 90 min of hands-on practice.	Diagnostic Acc: 0.49 (0.22) for physical examination; 0.75 (0.28) for HHUS (*p* < 0.001). LV systolic dysfunction: ↑ Se (25.9% vs. 74.1%); ↑ Sp (84.9% vs. 93.6%).
Mai et al., 2013 [54]	An expert cardiologist offered real-time remote audio-visual guidance for HHUS image acquisition and interpretation.	Adequate images in 90% of the patients retrieved by inexperience examiners vs. 96% by sonographers (*p* < 0.05).
Bonnafy et al., 2013 [31]	Three 3-h HHUS sessions (theoretical and practical training) to identify the Ao and adjacent structures and to measure Ao abdominal diameter. A comparison with CUS was carried out by experts.	Interoperator variability for experts (CUS) vs. novices (HHUS) was less than 4 mm in 92% of cases. No learning curve noted over time.
Gogalniceanu et al., 2010 [29]	Seminar, practical demonstration, case-based discussion, problem-solving exercise, 2 h of hands-on practice (HHUS and CUS), and 1 h assessment and feedback questionnaire.	A total of 85% of students completed a FAST scan with adequate views (mean score: 86%). A total of 96% of students preferred CUS (better image quality).
Swamy et al., 2012 [30]	Practical dissecting sessions: 15–20 min demonstration on a volunteer followed by 10–15 min hands-on training	A total of 40% of the students ↑ confidence in identifying anatomical structures, as well as a self-assessed improvement in the understanding of anatomy (*p* = 0.003).
Cawthorn et al., 2014 [32]	First year: 8 weeks (2 h weekly), 6 1-h practical sessions. Third year: (a) four 2-h lectures for 2 weeks; (b) 3 interactive online e-learning modules; (c) practical image acquisition: Two-hour supervised hands-on training sessions, OR 2 self-directed two-hour simulation-based scanning sessions, OR unlimited self-directed practice with HHUS over 4 weeks.	A total of 85% improvement (1st year). A total of 137% mean improvement in image interpretation (3rd year). Self-directed learning is not as efficient as supervised training sessions for gaining image acquisition skills.
Andersen et al., 2014 [33]	Nine hours of theoretical and practical training, as well as hands-on sessions by specialists. Students used their own HHUS devices and performed at least 75 exams.	Interpretable images: 73.8% of cases for cardiovascular structures; 88.4% for abdominal and pelvic images. Diagnostic Acc: 93% (abdominal Ao aneurysm and free abdominal fluid); 87.6% (gallbladder pathology).
Stokke et al., 2014 [34]	Four hours of focused ultrasound training. Sixty minutes of performing examinations on each other and seventy-five minutes of practicing in the cardiology ward.	↑ Se from 29% to 69% (*p* < 0.001) for mitral regurgitation. ↑ Se (*p* > 0.05) for Ao stenosis and regurgitation. Sp > 89% for all valvular diagnoses. Se = 25% for Ao root diameter.
Yan et al., 2015 [35]	Six hours of echocardiography training using parasternal long-and short-axis, apical four-chamber, and subcostal views in B- and color Doppler-mode to detect significant* valvular lesions.	US was superior to PE: significant MS, Ao stenosis and TR. Interobserver agreement: 0.12 after 2 weeks → 0.42 after 4 weeks. No improvement in physical examination (PE).
Kapur et al., 2016 [36]	Three sessions of theoretical and intensive hands-on training (12–15 h per student performed on mannequins and volunteers).	Knowledge improvements for normal US images of abdominal organs (*p* < 0.05); 98% of participants want similar programs regarding clinical training.
Yan et al., 2018 [37]	Six hours of training carried out by a cardiologist (two hours on US anatomy, one hour on case studies, and three hours of hands-on training). Comparison with transthoracic echocardiographies carried out by experts.	HHUS makes students more proficient at identifying valvular lesions (κ = 0.45) compared to physical examination (κ = 0.28) (*p* < 0.01).
Galusko et al., 2018 [38]	A total of 40 students (in groups of 6–10) participated in compulsory courses on cardiac murmurs, with limited exposure to HHUS (25 min/group). Six students participated in a 3-week extra-curricular HHUS echocardiography course.	Compulsory session—not all students managed to get hands-on experience with the Vscan. Extra-curricular HHUS course: the ability to recognize basic anatomy improved from 40% to 82% (*p* = 0.027); the ability to recognize valve disease and impaired LV ejection fraction increased from 4.2% to 71%.
Jerg et al., 2018 [39]	Four weeks of clinical traineeship for up to four people (HHUS for RUSH protocol).	~90% of students described the use of HHUS devices as helpful in improving their examination skills.
Nausheen et al., 2019 [40]	Four hands-on ultrasound sessions (gastrointestinal and renal systems).	Students were able to identify the liver (60%) and spleen (40%) without help; they identified the major branches of the Ao (30%), only two branches of the Ao (40%), the portal vein (30%), and the bile duct (30%) with guidance. A total of 30% could not locate any branches of the Ao, and all students needed help to locate the gall bladder and pancreas.
Nausheen et al., 2020 [42]	Confidence and ability to perform abdominal US. Goodman and Kruskal’s gamma was applied to assess the students’ abilities and their self-reported confidence levels.	Generally, self-confidence levels were low. Overall, 13–20% of students felt “very confident” about performing an ultrasound. Almost 37% needed encouragement and support, and almost 10% of the students were not willing to try to locate difficult organs.
Bar et al., 2021 [51]	Patients with suspected pneumothorax underwent ultrasound in two points of each hemithorax. Sensitivity and specificity for pneumothorax diagnosis by ultrasound and physical examination were calculated and compared with chest CT.	Ultrasound: 95.6% sensitivity and 97.44% specificity. Chest x-ray had the lowest sensitivity (47.8%) for pneumothorax detection. Physical examination showed a moderate sensitivity and specificity (82.6% and 77.89%).
Kameda et al., 2022 [41]	The participants were randomized into a video lecture or self-training group and completed pre- and post-tests.	The written post- vs. pre-test total scores were significantly higher. The combination of the video lecture and telepresence instructions was perceived as effective preparation for the post-test.
Kaiser et al., 2022 [43]	Ultrasound practical course with integrated hands-on activity using Vscan Air™, including duplex mode. The quality of the ultrasound images from previously selected organs was evaluated.	The rated image quality (18) never fell below a mean of 3 for the examined organs (liver, spleen, kidneys, rectovesical space, portal vein, pancreas, hepatic artery, and veins).
Edwards et al., 2022 [46]	Five ultrasound-teaching sessions—anatomy education. Five-point Likert questionnaire on self-perception.	HHUS anatomy education: improved understanding (93%); ↑ understand of the clinical relevance of learning anatomy (94%). Overall, 97% enjoyed the sessions, and 95% believed that US should be in the curriculum.
Acheampong et al., 2022 [52]	Trained to perform echocardiography (Gana). A total of 50 echocardiograms and 20 interpretations.	High agreement across all aspects: LV and RV structure and function and presence of effusion.
Jujo et al., 2022 [44]	Students took five-view echocardiographic image acquisition skill tests at pre-, immediate post-, and 8 weeks post-training. Three blinded assessors rated the image (max 10 points).	Medium-term skill retention was reported. The curriculum was sufficient for subcostal four-chamber and subcostal inferior vena cava skill retention but inadequate for parasternal long axis, parasternal short axis, and apical four-chamber.
Slader et al., 2022 [45]	Two days of instruction on core ultrasound fundamentals on two groups: handheld devices vs. standard US. Qualitative survey regarding personal experience. Overall, 604 images were obtained and graded by two emergency medicine physicians.	No statistically significant difference in participants’ perceived difficulty of ultrasound learning, comfort level, or self-estimated capability to perform ultrasounds in the future. The median quality score of the images obtained by the standard group was eight compared to seven in the handheld group (*p* < 0.01).
Bui et al., 2023 [47]	One-hour Zoom POCUS education program and in-person training (urology), and two weeks independent practice. Assessment of ultrasound competency and participant satisfaction.	The written exam scores were 3.36/5 in the training phase. Students expressed satisfaction with the program.

Value (value) = arithmetic mean (standard deviation); HHUS = handheld ultrasound; Acc = accuracy; LV = left ventricle; Se = sensibility; Sp = specificity; Ao = aorta; RUSH = Rapid Ultrasound in Shock and Hypotension; PE = physical examination; significant * = moderate to severe; MS = mitral stenosis; TR = tricuspid regurgitation. ↑ = increase.

**Table 4:** Quality of reporting assessment with MERSQI (Medical Education Research Study Quality Instrument).

Domain	MERSQI Item	MERSQI Sub-Item	No (%)
Study design	Single-group cross-sectional or single group post-test only		13 (50.0)
	Single-group pretest and posttest		8 (30.8)
	Non-randomized (two or more groups)		3 (11.5)
	Randomized controlled trial		2 (7.7)
Sampling	No. of institutions studied	1	23 (88.5)
		2	1 (3.8)
		>2	1 (3.8)
	Response rate percentage	<50% or not reported	22 (84.6)
		50–74%	0 (0)
		>75%	1 (0)
		Not applicable	3 (11.5)
Type of data	Assessment by study participant (knowledge self-report)		7 (26.9)
	Objective measurement (knowledge test)		20 (76.9)
Validity of evaluation instrument	Internal structure	Not applicable	3 (11.5)
		Not reported	23 (88.5)
		Reported	0 (0)
	Content validity	Not applicable	3 (11.5)
		Not reported	23 (88.5)
		Reported	0 (0)
	Relationships to other variables	Not applicable	3 (11.5)
		Not reported	23 (88.5)
		Reported	0 (0)
Data analysis	Appropriateness of analysis for study design or type of data	Inappropriate	2 (7.7)
		Appropriate	24 (92.3)
	Complexity of analysis	Descriptive analysis only	9 (34.6)
		Beyond descriptive analysis	17 (65.4)
Outcomes	Satisfaction, attitudes, perceptions, opinions, general facts		5 (19.2)
	Knowledge, skills		19 (73.1)
	Behaviors		1 (3.8)
	Patient/healthcare outcomes		3 (11.8)

## Data Availability

All data supporting reported results can be found in the article.
